# A “rolling average” multiple adaptive planning method to compensate for target volume changes in image‐guided radiotherapy of prostate cancer

**DOI:** 10.1120/jacmp.v13i1.3697

**Published:** 2012-01-05

**Authors:** Han Liu, Qiuwen Wu

**Affiliations:** ^1^ Department of Radiation Oncology Duke University Medical Center Durham North Carolina 27710 USA

**Keywords:** prostate cancer, adaptive radiotherapy, image‐guided radiation therapy, intensity‐modulated radiation therapy, margins

## Abstract

For prostate cancer radiotherapy, the interfractional organ motion can have several forms: changes in position, shape, and volume. The interfractional motion can be managed through either online or offline image guidance (IG). The position changes are commonly corrected through online IG by correcting couch position at each treatment fraction, while the shape and volume changes, or target deformation, can be compensated by margins in offline adaptive planning. In this study, we proposed and evaluated a rolling‐average (RA) adaptive replanning method to account for the target volume variations. A total of 448 repeated helical computed tomography (HCT) scans from 28 patients were included in the study. Both low‐risk patients (LRP, CTV=prostate) and intermediate‐risk patients (IRP, CTV=prostate + seminal vesicles) were simulated. The benefit of RA strategy was evaluated geometrically and compared with the standard online IG‐only method and a single replanning adaptive hybrid strategy. A new geometric index, cumulative index of target volume (CITV), was used for the evaluation. Two extreme scenarios of target volume changes, Type Ascending and Descending, were simulated by sorting the CTV volumes of actual patient data in order to have a better evaluation of the methods. Modest target volume variations were observed in our patient group. The prostate volume change was −0.14±0.11 cc/day (or −0.30%±0.26% per day). It is found that RA is superior to the online IG and hybrid techniques. However, the magnitude of improvement depends on how significantly and rapidly the target volume changes. On the issue of planning complexity, the hybrid is more complex than online IG only, requiring one offline replanning, and RA is significantly more complex, with multiple replanning. In clinical implementation of RA, the effectiveness and efficiency should be balanced. The effectiveness is dependent on the patient population. For low‐risk patients, RA is beneficial if there is significant time trend in target volume during the treatment course of radiotherapy. The optimal number of fractions necessary for the internal target volume (ITV) construction is 2 for LRP and 3 for IRP for RA strategy.

PACS numbers: 87.55.D‐; 87.55.de

## I. INTRODUCTION

A major concern in fractionated radiation therapy (RT) of prostate cancer is the uncertainties from setup errors and interfractional organ motions. If not taken into account properly, they can affect the accuracy of the treatment, and the dose delivered to the target volume may be quite different from the prescribed dose on the planning CT image. The setup error correction has been covered by many studies,^(^ ^1^ ^,^ ^2^ ^)^ and can be implemented through either online or offline image guidance (IG).^(^ ^3^ ^,^ ^4^ ^)^ Uncertainties caused by target motion are more complicated. The position and shape changes of prostate and seminal vesicles (SVs) are affected by physiological status of fullness of the bladder and rectum.^(^ ^5^ ^,^ ^6^ ^)^ Similar to setup error correction, both online and offline IG strategies have been used to manage organ motion.^(^ ^3^ ^,^ ^7^ ^,^ ^8^ ^)^ The interfractional organ motion can have several forms: changes in position, shape, and volume. Currently the most common form of prostate image‐guided radiotherapy (IGRT) is online IG, in which daily images of the patient anatomy is acquired and aligned with the planning image and, as a result, the changes in position of the target is corrected. The shape and volume changes, or target deformations, are typically handled offline through the use of margins or adaptive planning.^(^ ^3^ ^,^ ^9^ ^)^ This paper addresses the third factor of the interfractional motion noted above: change in volume.

During a treatment course of prostate cancer RT, noticeable volumetric changes can occur for both the target and critical organs. The reasons for the volume variation include response to radiation, hormonal therapy, or combination of both. There are considerable controversies in the volumetric trend results. The shrinkage of prostate volume during the treatment course of RT has been reported by a few studies with computed tomography (CT) scans.^(^ ^10^ ^–^ ^12^ ^)^ In some studies, no time trend was observed in prostate volume using CT scans,^(^ ^6^ ^)^ and MRI images.^(^ ^13^ ^)^ A study by Nichol et al.^(^ ^14^ ^)^ observed that the prostate volume increased by as much as 34% in the early course of RT, and decreased by as much as 24% in the late course of RT. For intermediate‐risk and high‐risk prostate cancer patients, SVs are usually included in the clinical target volume (CTV) for the treatment.^(^ ^15^ ^)^ It was noted that the SV volume could vary by as much as 100% during the treatment course of RT.^(^ ^6^ ^)^ However, no such significant volumetric change in SV was observed in a recent study.^(^ ^12^ ^)^ There are many possible reasons for such a large discrepancy: variation in patient cohort, patient response to change in treatment fractionation and treatment duration, and the effect of hormonal therapy. None of these studies provides conclusive evidence about the changes in prostate and SV volumes during the course of RT. Furthermore, only one or a few images were acquired during the treatment course for analysis. More frequent images are necessary in order to assess detailed information of volumetric variations of prostate and SVs with time.

If a significant change in the target volume occurs during the treatment course, the treatment plan based on the initial planning image may not be optimal for the changing anatomy throughout the treatment. This is the case even after various image‐guidance corrections have been applied, because all current image‐guidance techniques do not take into account the change in target volume. This may lead to large discrepancies between the actual delivered dose to the patient and the planning dose, and thus result in significantly increased dose to normal structures and critical organs. One technique to account for the patient anatomic changes is to re‐optimize the treatment plan based on the images acquired in previous treatment fractions. For example, it was found that adaptive replanning to account for the tumor shrinkage is beneficial for improved sparing of critical organs for head and neck RT.^(^ ^16^ ^)^ As far as prostate cancer is concerned, to date there has been no similar study on the adaptive replanning for volumetric change. The purposes of this study are: (1) to analyze the time trend of the volumetric changes of prostate and SVs during the treatment course of RT; (2) to propose a rolling‐average (RA) adaptive replanning method to account for the volume changes; and (3) to evaluate the effectiveness of the RA method by comparing with the standard online image guidance only strategy and a single replanning hybrid strategy.

## II. MATERIALS AND METHODS

### A. Patient data and time trend analysis

Repeated helical computed tomography (HCT) images of the pelvic region from 28 prostate cancer patients were selected in this study. Each patient had one planning HCT scan and at least 15 HCT scans (3 mm slice thickness) with a conventional helical scanner during the treatment course. All CT images were imported into the Pinnacle treatment planning system (Pinnacle v8.0, Philips Radiation Oncology System, Madison, WI) for further processing. To remove the setup error, all treatment CTs of the same patient were rigidly registered to the planning CT based on the bony structures. The contours for prostate and SVs were delineated by experienced planners for each CT. The treatment for both low‐risk patients (LRP) and intermediate‐risk patients (IRP) were simulated. The CTV was prostate gland only for LRP, and prostate plus SVs for IRP. A hypofractionation protocol was used to simulate the treatment. The prescription dose was 3.9 Gy per fraction to the prostate over 15 fractions, for a total dose of 58.5 Gy. Assuming α/β=4, the prescription dose was biologically equivalent to 80 Gy treated at 1.8 Gy per fraction, with a minimum dose to prostate of 76 Gy.

The original HCT image had the resolution of ~1 mm×1 mm×3 mm. To avoid the round off error by the CT slice thickness, both CT images and contours were interpolated into 1 mm thick slices in the superior–inferior direction (as proposed in a paper by Lei and Wu^(^ ^9^ ^)^), so the precision becomes ~1 mm in all directions. This is also the precision that can be reasonably achieved by typical image‐guidance systems, such as the treatment couch. All the following studies were based on the interpolated images and contours.

The volumetric variations over treatment time for both prostate and SVs were analyzed by using linear regression analysis for each patient. In order to assess volume time trend among different patients who may have different imaging schedules, we interpolated the prostate volumes across the treatment days. Treatment days were computed as:

(1) Days=Gap+(i−1)* Interval

where *Gap* is the time interval between planning CT and the first treatment fraction, *Interval* is the time duration between treatment fractions, and *i* (i=1,2,…, 15) is the treatment fraction number. It was noted that for the group of patients we analyzed, there was about a one‐week time (Gap) between the planning CT and the first treatment fraction, and the last (15th) treatment CT images were acquired between 49 and 70 days after the planning CT, with a mean value of 53.1 (± 4.7) days. So in this study, we chose Gap=7 days and Interval=3 days, and the volume averaging was limited to 49 days after the planning CT. If the CT image for a patient was not available for that day, the volume was interpolated based on the neighboring CT images. Under such arrangement, all patients can be included in the analysis at the cost that some measured data were not used.

Furthermore, in order to reduce the contouring uncertainties and have a better evaluation of the adaptive planning methods, two extreme scenarios were simulated by rearranging the interpolated volumes across the treatment fractions: (1) Ascending Type, by which the target volumes were sorted from the smallest to the largest for each patient; and (2) Descending Type, by which the volumes were sorted from the largest to the smallest.

### B. Online image‐guidance and offline adaptive replanning strategies

**Figure 1:**
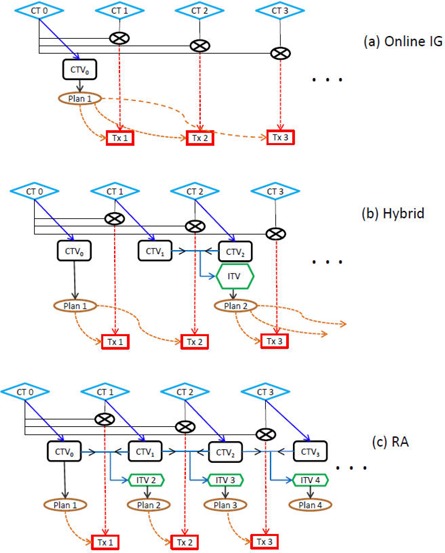
Work flow for: online IG only (a), hybrid (b), and RA (c) strategies. For illustration, the number of fractions used for ITV construction was chosen to be 2 for both the hybrid and RA strategies. The symbol ⊗ represents online registration between treatment CT and planning CT images.

Figure 1(a) depicts the typical online image‐guidance procedure. The planning CT image (CT 0) is acquired at the day of CT simulation. Contours of ${\text{CTV}}_{0}$ and other organs are delineated and the treatment plan (Plan 1) is generated offline. At each treatment fraction, the treatment CT image (CT i, i=1…15) is acquired, image registration is performed between the planning CT (CT 0) and treatment CT (CT i), followed by patient position correction, and treatment is delivered afterwards with Plan 1.

For both LRP and IRP, the online registration was simulated by matching the center of mass (COM) of the prostate from each treatment CT with those from the planning CT proposed by Liang et al.^(^ ^17^ ^)^ This registration was used to simulate online image guidance based on implanted markers in the prostate gland. Registration was limited to translations only in this study. The translational components in setup error and organ motion were assumed to be removed after this online correction.

The hybrid strategy procedure (a combination of a single offline adaptive replanning and online image guidance) was described by Lei and Wu.^(^ ^9^ ^)^ Here only a brief summary is given. To simulate the hybrid strategy, a few fractions early in the treatment course were used to construct a new target volume, the internal target volume (ITV), for each patient:

(2) $${\text{ITV}}_{n}=\overset{n}{\underset{i=1}{\cup }}{\text{TM}}_{i}[{\text{CTV}}_{i}],\;\;\;\;(n=2,3,4),$$

where ${\text{TM}}_{i}$ is the translational transformation matrix used in online IG of the i‐th treatment fraction, ${\text{CTV}}_{i}$ is CTV at the i‐th fraction, and *n* is the number of fractions used in the ITV construction. The workflow of the hybrid strategy is illustrated in Fig. 1(b) for n=2. An initial Plan 1 based on ${\text{CTV}}_{0}$ is created and used for treatment delivery of fractions 1 and 2 with online image guidance. Contours of ${\text{CTV}}_{1}$ and ${\text{CTV}}_{2}$ are drawn offline on treatment images CT 1 and CT 2, and an ITV is constructed by the unions of ${\text{CTV}}_{1}$ and ${\text{CTV}}_{2}$. A modified Plan 2 is created based on the ITV and applied for treatment delivery of future fractions. Compared with online image guidance only, the hybrid strategy is more complex, in that the contouring and replanning actions are taken between treatment fractions. However, there is no effect on the online treatment efficiency.

In this study, we propose a new adaptive planning method, the rolling‐average multiple replanning strategy, or RA (a combination of *multiple* offline adaptive replanning and online image guidance). The internal target volume for the i‐th treatment fraction is constructed based on a few prior treatment fractions:

(3) $$\{\begin{array}{c} {\text{ITV}}_{1}={\text{CTV}}_{0}\;\;\text{for}\;\;i=1\\ {\text{ITV}}_{i}={\text{CTV}}_{0}\cup \overset{\text{min}(i,n)}{\underset{j=0}{\cup }}{\text{TM}}_{j}({\text{CTV}}_{j})\;\;\text{for}\;\;1<i<n.\\ {\text{ITV}}_{i}=\overset{i-1}{\underset{j=i-n}{\cup }}{\text{TM}}_{j}({\text{CTV}}_{j})\text{for}\;\;\;i\geq n \end{array}$$

where n=2,3,4 is the number of fractions used to construct the ITV. The work flow of RA strategy is illustrated in Fig. 1(c) for n=2. An initial Plan 1 is produced based on ${\text{CTV}}_{0}$ and used only for treatment delivery of the first fraction Tx 1. After each fraction, a new treatment plan is produced based on a new ITV, which is formed by the unions of two most recent CTVs prior to that fraction.

Both the hybrid and RA strategies handle the volume and shape changes in the same manner; however, the efforts spent offline for the ITV construction and replanning are different. For the hybrid strategy, only a single ITV is constructed and a single replanning is performed at the beginning of treatment course. For the RA strategy, the ITV differs from fraction to fraction, and multiple replanning procedures are performed. When one recent CTV is added to the new ITV construction, the most distant CTV is dropped from ITV. The hybrid strategy cannot reflect the volume variation throughout the course of treatment since only a few early CTVs are used for the ITV construction. However, with the RA strategy, by dropping the early CTV in the ITV construction, one can in principle minimize the effect of large systematic volume changes.

An extreme case which uses all previous available information was also investigated in this study: ${\text{RA}}_{\text{infinity}}$ strategy. The ITV for ${\text{RA}}_{\text{infinity}}$ can be expressed as:

(4) $$\{\begin{array}{c} {\text{ITV}}_{1}={\text{CTV}}_{0}\;\;\;\;\;\text{for}\;\;\;\;\;i=1\\ {\text{ITV}}_{i}=\overset{i-1}{\underset{j=0}{\cup }}{\text{TM}}_{j}({\text{CTV}}_{j})\;\;\;\;\;\text{for}\;\;i>1 \end{array}$$

### C. Cumulative index of target volume to evaluate image‐guidance strategy

The volume overlap index (OI) was used for the geometric evaluation. A range of uniform margins (0–10 mm with 1 mm increments) were added to the CTV (or ITV depending on the strategy) to form the PTV. For each margin value, the overlap index between the PTV and subsequent CTV at each treatment fraction after online image guidance can be computed. The margins required for different strategies to achieve 99% of average overlap index were interpolated from the relations between the average OI and margin added. The benefit of RA over hybrid and online IG only can be evaluated by comparing the margin added volume among different strategies when they have equal overlap index.

In a recent study, a new geometry‐based index, cumulative index of target volume (CITV), was proposed for the evaluation of different image guidance strategies:^(^ ^18^ ^)^

(5) $$CITV=\frac{\Sigma_{i}\;Volume\;(PTV_{i})}{\Sigma_{i}\;Volume\;(CTV_{i})},\;\;\;\;\;\;\;(i=1,2,\;\ldots ,\;N),$$

where *N* is the total number of treatment fractions. In standard radiotherapy, only a single PTV is used in a single plan; the numerator becomes the PTV multiplied by the total number of fractions. For adaptive radiotherapy, multiple plans may exist and PTV may be different at each fraction. For example, in hybrid strategy, we require that ${\text{PTV}}_{i}={\text{CTV}}_{i}+M$, where *M* is the margin added to achieve average 99% of overlap index. The CITV has a few advantages over other indices used today. It is geometric in nature, and has a simpler scheme than the geometric margin because there is no need to include additional margin to account for the volume difference between ${\text{CTV}}_{0}$ and the ITV, and it covers the effect of the entire treatment process. Furthermore, CITV is based on three‐dimensional volume, so it can be expanded to compare strategies using nonuniform margins. In addition, CITV can be applied for sub‐mm comparisons, and by definition it has the accumulation effect built in, while most other current geometrical indices do not. Since the CITV is not in a dosimetry domain, it does not depend on the parameters used in the treatment planning, such as photon energies, number of beams, beam angles, or treatment modalities. It is only a function of the underlining patient motion uncertainties. Therefore, it can be generalized and easily adaptable to different clinics where the treatment planning process can be quite different.

The CITV was used for the evaluation of different planning methods in this study. For the online IG only strategy, let $M_{1}$ be the margin required to achieve OI=99%; PTV is constructed by expanding ${\text{CTV}}_{0}$ with margin $M_{1}$: $\text{PTV}={\text{CTV}}_{0}+M_{1}$. CITV can be simplified as:

(6) $$CITV(\text{Online}\;\text{IG})=\frac{N\times Volume\;(PTV)}{\Sigma_{i}\;Volume\;({\text{CTV}}_{i})},\;\;\;\;\;\;\;(i=1,2,\;\ldots ,\;N).$$

For the hybrid strategy, we have ${\text{PTV}}_{1}={\text{CTV}}_{0}+M_{1}$ for i≤n (n=2,3,4 is the number of fractions used to construct the ITV) and ${\text{PTV}}_{2}={\text{ITV}}_{n}+M_{2}$ for i>n. CITV can be rewritten as

(7) $$CITV(\text{Hybrid})=\frac{n\times Volume\;(CTV_{0}+M_{1})+\left(N-n\right)\times Volun}{\Sigma_{i}\;Volume\;({\text{CTV}}_{i})}$$

$M_{2}$ is the margin required to obtain OI=99% for the hybrid strategy.

Similarly, for the RA strategy, ${\text{PTV}}_{i}={\text{ITV}}_{i}+M(i=1,2,\;\ldots ,\;N)$, where *M* is the average margin needed to obtain 99% of OI. CITV takes the form:

(8) $$CITV(\text{RA})=\frac{\Sigma_{i}\;Volume\;(ITV_{i}+M)}{\Sigma_{i}\;Volume\;({\text{CTV}}_{i})},\;\;\;\;\;(i=1,2,\;\ldots ,\;N).$$

There are some disadvantages to using real patient data when evaluating the benefit of the RA strategy. For example, there are contouring errors, represented by the random variations of the target volume with time, or the time trend in target volume may not be significant. In order to have a better evaluation of the methods, two extreme cases were simulated based on the CTV volumes of actual patient data (Type Normal): (1) Type Ascending: the volume of CTV was sorted from the smallest to the largest, (2) Type Descending: the volume of CTV was sorted from the largest to the smallest. The systematic changes in these two types can test whether the adaptive planning method is suitable for such changes.

Four margin designs were investigated in this study:

Margin I: patient‐specific fractional margin, which can be calculated by interpolating from the relationship between OI and margins added to the CTV (or ITV) to achieve 0.99 average OI for each patient;

Margin II: patient‐specific integer margin, the nearest integer greater than or equal to the fractional margin for each patient, a value that is conservative and yet achievable by many planning systems;

Margin III: patient population‐based fractional margin, the mean value of patient‐specific fractional margin from all patients; and

Margin IV: patient population‐based integer margin, the nearest integer greater than or equal to Margin III.

The Margin III is applicable uniformly to all patients and yet has higher precision than its integer counterpart; therefore it is best suited for this study, and only results based on this type of margin are presented in detail. Studies based on other types of margins were also performed but results are not presented. The rational and difference between them are presented in the discussion.

## III. RESULTS

### A. Volumetric change over time for prostate and seminal vesicles

**Figure 2:**
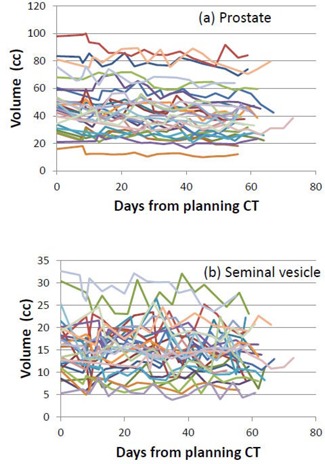
Volume variations for each patient over treatment time for: prostate (a) and seminal vesicles (b).

Sixteen repeated HCT scans for each patient allowed us to assess detailed information on time trend in prostate and SV volumes during the course of treatment. The volume variations over treatment time for each patient are presented in Figs. 2(a) and (b) for prostate and seminal vesicles, respectively. The prostate volume from the planning CT was 48.7±19.3 cc. Modest target volume variations were observed in our patient group. The change in prostate volume was −0.14±0.11 cc/day, or −0.30% ±0.26 per day. Linear regression analysis showed that for half of the patients (14 out of 28 patients), there were statistically significant shrinkage in prostate volume with time (p>0.05). The volume variation for this group of patients was −0.18±0.10 cc/day, or −0.39% ±0.25 per day with a range from −0.39 to −0.02 cc/day, or −0.88% to −0.03% per day. For the other half of the patients, the time trend was not statistically significant, either decrease or increase. The variation in prostate volume was −0.22±0.24 cc/day, or −0.10% ±0.10 per day with range from −0.22 to 0.09 cc/day or −0.64% to 0.19% per day. Typically SVs have relative small volume compared with prostate. The volume of SVs for planning CT was 16.2±6.3 cc. Only three patients showed significant shrinkage, and one patient showed significant growth in SV volumes during the course of treatment.

**Figure 3:**
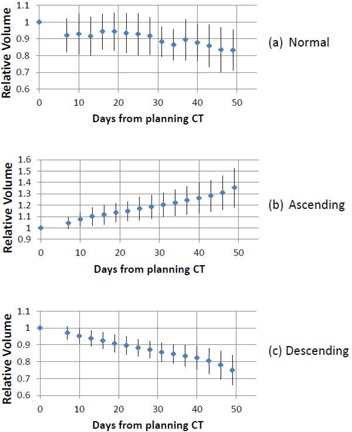
Variations of interpolated prostate volume with treatment time for: type Normal (a), type Ascending (b), and type Descending (c).

The average interpolated prostate volumes over all patients for each treatment day were normalized to that of the planning CT for real data (type Normal), type Ascending and Descending, respectively, and the results are shown in Fig. 3 as a function of the elapsed treatment days. Linear regression analysis was performed and significant time trends were observed for all three types with p<0.0001. The coefficients of determination, $R^{2}$, from the regression analysis were 0.89 for type Normal, and 0.99 for both type Ascending and Descending. Both type Ascending and Descending had more significant time trend than that of type Normal, which was expected according to how we simulated the data.

### B. Geometric evaluation for different image guidance strategies

In a previous study, it was demonstrated that the optimal number of treatment fractions necessary for the ITV construction for hybrid strategy is 2 for LRP, and 3 for IRP.^(^ ^18^ ^)^ Therefore, only n=2 for LRP and n=3 for IRP of hybrid strategy are presented in this paper for comparison.

The population‐based uniform margins required to have an average overlap index of OI=0.99 were derived from the relationship between the OI and margins added to the ${\text{CTV}}_{0}$ (or ITV), and they are summarized in Table 1 for different IG strategies, and for both LRP and IRP. From the table, we can see that:

(1) Margins for IRP are larger than that of LRP for all image‐guidance strategies, mainly due to the irregular shape of the CTV in IRP and large interfractional motion of SV.
(2) For hybrid and RA strategies, the planning margins required can be reduced for both LRP and IRP, compared with the standard online IG only strategy. However, this margin difference should be discounted due to the volume difference in ${\text{CTV}}_{0}$ and ITV.
(3) For same n of hybrid and RA strategies, M(Ascending)>M(Normal)>M(Descending) for LRP. This is understandable because type Ascending had the smallest ${\text{CTV}}_{0}$ volume while type Descending had the largest ${\text{CTV}}_{0}$ volume.
(4) For same n, M(Hybrid, Ascending)>M(RA,Ascending) and M(Hybrid, Descending)<M(RA,Descending). This is because only a few early fractions were used in ITV construction for hybrid strategy, while for RA strategy only a few most recent fractions were used.
(5) Margins decrease with the increasing number of fractions used in ITV construction for both hybrid and RA strategies. However, the actual margin difference should be smaller due to the volume differences between ITVs.
(6) The RA infinity strategy yields the smallest margin for both groups of patients, mainly because all the previous measurements were taken into account in the new ITV construction.

**Table 1:** The uniform margins (mm) needed to obtain an average overlap index of 0.99 for different image‐guided strategies.

*Group*	*Method*	*Number of fractions*	*Ascending*	*Margins (mm) Normal*	*Descending*
LRP	Online IG	‐	4.4±1.1	2.6±0.9	2.0±0.8
	Hybrid	2	2.9±0.9	1.7±0.6	1.0±0.5
		2	2.1±0.7	1.8±0.5	1.6±0.6
		3	1.7±0.6	1.4±0.5	1.2±0.5
	RA	4	1.6±0.7	1.1±0.4	1.0±0.4
		Infinity	‐	0.9±0.4	‐
IRP	Online IG	‐	5.5±1.6	4.7±1.8	4.1±2.2
	Hybrid	3	3.2±1.3	2.1±1.0	1.5±0.8
		2	3.3±1.2	3.1±1.4	3.1±1.5
	RA	3	2.7±1.0	2.4±1.1	2.4±1.3
		4	2.4±0.9	2.2±1.1	2.0±1.2
		Infinity	‐	1.7±1.1	‐

LRP=low−risk patients; IRP=intermediate−risk patients; Online IG=online image guidance only strategy; Hybrid=online image guidance and offline adaptive single replanning strategy; RA=online image guidance and rolling average multiple replanning strategy; Ascending=target volumes sorted from the smallest to largest; Normal=real patient data; Descending = target volumes sorted from the largest to smallest.

Since the ITV was the union of ${\text{CTV}}_{i}$ from a few previous treatment fractions, the volume of ITV was usually larger than that of ${\text{CTV}}_{0}$. Comparing the margins alone can lead to incorrect conclusions due to the volume difference between ITV and ${\text{CTV}}_{0}$. In order to have a fair comparison of different image‐guidance strategies, additional margin needs to be included to account for the volume difference. To avoid these complicated and approximate calculations, we will use CITV for the evaluation of different image guidance strategies in this study.

**Figure 4:**
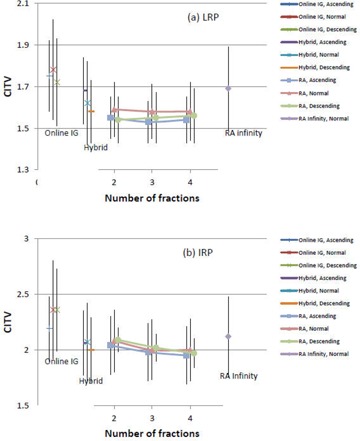
Cumulative index of target volume (CITV) using population‐based fractional margin for the online IG only, hybrid and RA strategies: low‐risk patients (a), intermediate‐risk patients (b). The symbols on the left are for the online IG only and hybrid strategies; the rest are for the RA strategy. Vertical scales are different for LRP and IRP.

The cumulative index of target volume is shown in Fig. 4 for online IG only, hybrid, and RA strategies for both LRP and IRP. From the figure we observe the following:

(a) For online IG only and same n with hybrid and RA strategies, CITV(LRP) < CITV(IRP) (CITV(LRP)<CITV(IRP) from paired Student's t‐test), which means cumulative relative target volumes get irradiated are significantly smaller for LRP than those for IRP.
(b) For both groups of patients:
  - The CITV for the online IG strategy is the largest (Student's t‐test p<0.0001), indicating that both the hybrid and RA strategies are better than the online IG only strategy. (For LRP, CITV(online IG) is 10% and 12% higher than CITV(Hybrid) and CITV(RA). For IRP, the differences are 19% and 23%, respectively).
  - For the same n, CITV(RA)<CITV(Hybrid), suggesting that the RA strategy is better than the hybrid strategy. However, the magnitudes of improvement are small, which is statistically insignificant at 2% for LRP, and statistically significant at 4% for IRP.
  - CITV(RA,Infinity)>CITV(RA, n=2,3,4) by 6% with p<0.01 from paired Student's t‐test. Even though the margins required to achieve 99% of overlap index for ${\text{RA}}_{\text{infinity}}$ strategy were the smallest, the ITV volumes for later treatment fractions were larger and larger due to the increasing number of fractions used for ITV construction. Accordingly, the PTV volumes for later fractions were larger and larger in the ${\text{RA}}_{\text{infinity}}$ strategy, and resulted in larger numerator values in Eq. (8) for the ${\text{RA}}_{\text{infinity}}$ than those of the RA with smaller n. Since the denominator in Eq. (8) does not depend on n, CITV(RA,Infinity)>CITV(RA, n=2,3,4). It was also noted that CITV(RA,Infinity)<CITV(online IG) by 5% for LRP and 16% for IRP, suggesting that the ${\text{RA}}_{\text{infinity}}$ strategy is better than the online IG only strategy.
(c) For LRP:
  - CITV(Ascending)>CITV( Normal)> CITV(Descending) for the hybrid strategy, which means that the hybrid strategy works better if the prostate volume decreases towards the end of treatment, and worse if the prostate volume increases during the treatment course.
  - For the RA strategy, CITV(Normal)>CITV(Ascending) and CITV(Normal)>CITV(Descending) (paired Student's t‐test p<0.001), suggesting that the RA strategy works better if there is a systematic time trend (either shrinkage or growth) in prostate volume during the treatment course.
  - For type Normal volume variation, the RA in general has lower CITV values than the hybrid. However, the difference is not statistically significant. For example, for patient 7, CITV(Hybrid)=1.40 is better than CITV(RA)=1.46; for patient 12, CITV(Hybrid)=2.38 is worse than CITV(RA)=1.94; for patient 20, CITV(Hybrid)=1.53 is same as CITV(RA).
(d) One way repeated measure ANOVA shows that there are significant differences in the CITV values between the online IG and RA strategies. The optimal number for ITV construction in the RA strategy is 2 for LRP, and 3 for IRP.

## IV. DISCUSSION

During the treatment course of radiation therapy, many patients develop significant anatomic changes in target volume due to many factors, including response to radiation and hormonal therapy. The anatomic changes of target can cause uncertainties in tumor localization and may potentially lead to large discrepancy between the planning dose and the actual delivered dose. The volumetric variation of the target can be effectively accounted for by adaptively modifying the treatment plan along the treatment course.

In this study, we performed geometric evaluation of the RA method. In principle, a comprehensive dosimetric study is necessary for thorough evaluation of the method. However, the dosimetric evaluation depends on many other factors such as the choice of photon energies, the beam arrangement, and the treatment modality. The result is usually a combination of the underlining patient organ motions and the choice of these parameters. In comparison, the geometrical evaluation is only dependent on the organ motions studied. Since the results showed that the geometric benefit of the RA over the hybrid strategy was relatively small, the dosimetric evaluation may not be necessary since studies showed that the dosimetric difference among image‐guidance strategies is usually less than the geometric difference.^(^ ^9^ ^,^ ^18^ ^)^ This is different from the investigation of the hybrid strategy, where both geometric and dosimetric evaluations were performed, because the benefit over the online image guidance alone is significant.

While only one type of interfractional organ motion was investigated in detail in this study, it should be realized that other types of uncertainties also exist during the treatment course, such as target definition error, residual of online image guidance, and intrafraction motion. The actual margins added during planning need to take all of them into consideration. As a result, the CITV values and the differences in CITV may be different.

### A. Comparison of different image guidance strategies

For the online IG and hybrid strategies, if isotropic target volume variation is the only uncertainty during the treatment course and full target coverage is required (i.e., OI=1), according to Eq. (5), we should have CITV(Ascending, OI=1)=CITV(Normal, OI=1)=CITV(Descending, OI=1). The reasoning is as follows: the denominators for all three methods are the same, and $V({\text{PTV}}_{i},\text{Ascending})=V({\text{PTV}}_{i},\text{Normal})=V({\text{PTV}}_{i},\;\text{Descending})=V(\text{max}({\text{CTV}}_{i}))$. If overlap index is allowed to be lowered to 0.99, then $V({\text{PTV}}_{i},\text{Ascending})<V({\text{PTV}}_{i},\text{Normal})<V({\text{PTV}}_{i},\;\text{Descending})=V(\text{max}({\text{CTV}}_{i}))$, we should have CITV(Ascending,OI=0.99)<CITV(Normal,OI=0.99)<CITV(Descending, OI=0.99)=CITV(Descending, OI=1). However, our results showed that CITV(Ascending, OI=0.99)>CITV(Descending, OI=0.99). This could not be explained by volumetric change alone, which is a strong indication that other factors such as target deformation may play important roles. It also suggests that for patients with large volume variation during the treatment, the plan quality for both the online IG and hybrid strategies is sensitive to the volume variations.

Among three image‐guidance strategies studied, the RA has the smallest CITV, and the online IG has the largest CITV for both groups of patients, indicating that both the hybrid and the RA strategies are better than the online IG only strategy, and the RA is better than the hybrid strategy. This is expected because more efforts are put into the RA through multiple replanning procedures. In addition, the RA is less sensitive to the volume variation when compared to the other two strategies. The more systematic changes in the volume, the better results the RA can achieve. However, as shown in Fig. 1, significant efforts are needed for the offline replanning for the RA strategy. The effectiveness and efficiency should be balanced. We would like also to point out that both the hybrid and RA do not affect the online IG and treatment efficiency. All the extra efforts and time are spent offline.

For LRP, the RA strategy works better if there is a significant time trend in prostate volume (either shrinkage or growth) during the treatment course. Similar statements cannot be made for IRP. The main reason, we believe, is that even the seminal vesicles have relative small volume compared with prostate; however, the large interfractional relative motion of the SV can cause large deformation of the whole CTV, and handling deformation is not a strength of RA.

The average volumes of the ITV are usually larger than that of the ${\text{CTV}}_{0}$ and increase with the number of fractions (*n*) used in the ITV construction for both the hybrid and RA strategies. However, the volumes of the PTV constructed from the ITV decrease and tend to saturate with the increasing *n* for both LRP and IRP. The optimal number of fractions necessary to construct the ITV in both the hybrid and RA strategies is 2 for LRP, and 3 for IRP, similar to hybrid strategy. Compared with the hybrid strategy, the benefit of cumulative irradiative target volume of the RA strategy may appear small in this study. However, other factors should be taken into consideration, such as how significant and rapid the volume varied during the treatment course. Only half of the patients in our study showed significant prostate volume changes, and the absolute volume change (on average about 8 cc from the plan CT to the end of treatment) seemed small. For patients with larger time trend and more rapid volume variations, the benefit of the RA strategy is expected to be more pronounced. Simply put, RA is no worse than other adaptive planning strategies.

### B. Impact of different margin designs

Only detailed results from Margin III (population‐based fractional margin) were presented in this paper. Beside Margin III, three other margin designs (I, II and IV) were also investigated for comparison. In the ideal situation, patient‐specific margin is the right choice for evaluation. However, it requires imaging information from all treatment fractions for each patient, thus is unrealistic for the adaptive planning. The patient population‐based integer margin is the most realistic choice due to its simple nature and the fact that most treatment planning systems cannot handle fractional and sub‐mm margins. We chose population‐based margin in our study because it can be applied to all patients, including future ones. There are many uncertainties in the entire treatment process of radiotherapy such as setup error and intrafractional motion. The margin to compensate for each independent uncertainty is usually investigated separately. The final overall margin is then the combination of these submargins added in quadrature. The overall margin will be overestimated significantly if only integer margins are used for each submargin determination. Therefore, the results from the population‐based fractional margin were selected for presentation in detail in this study. Except the magnitude of CITV values (CITV(Margin I) is about the same as CITV(Margin III), and both are less than CITV(Margin II)), no qualitative differences among Margin I, II, and III were observed. CITV(Margin II) are about 6%–10% more than CITV (Margin III). However, no similar conclusions can be drawn for Margin IV, primarily due to the actual values of population‐based fractional margin. In another words, the CITV values for Margin IV have a very strong dependence on how far the population‐based fractional margins are to the nearest integer towards infinity. We also would like to point out that, by definition, the integer margin designs always overestimate the total cumulative irradiated volume. It was noted that there were large standard variations for integer margin design compared with fractional one, either population‐based or patient‐specific.

## V. CONCLUSIONS

In summary, we have developed a “rolling average” adaptive planning method to account for the volume change and quantitatively evaluated its benefits. We have proved that the RA is beneficial; however, the magnitude of improvement is dependent on how rapidly the target volume changes. We observed modest target volume variations in our patient group. If the time trend is small, then the RA may not be necessary and the hybrid strategy may suffice. The hybrid strategy is more complex than online IG only, and the RA is significantly more complex. In short, there are two residual uncertainties in the interfractional organ motion after the online image guidance: shape change and volume changes. We believe that the hybrid is more effective for shape change and the RA is more effective in systematic time trend of target volumes.

## VI. ACKNOWLEDGEMENT

This study is partially supported by grant CA118037 from the National Institute of Health. The contents are solely the responsibility of the authors and do not necessarily represent the official view of the NIH. We thank Dr. Justus Adamson for proofreading the manuscript.
